# Synergistic N_2_-fixation and salt stress mitigation in soybean through dual inoculation of ACC deaminase-producing *Pseudomonas* and *Bradyrhizobium*

**DOI:** 10.1038/s41598-023-43891-4

**Published:** 2023-10-10

**Authors:** Khin Thuzar Win, Sawa Wasai-Hara, Fukuyo Tanaka, Aung Zaw Oo, Kiwamu Minamisawa, Yoshikazu Shimoda, Haruko Imaizumi-Anraku

**Affiliations:** 1 Institute of Agrobiological Sciences, National Agriculture and Food Research Organization (NARO), Tsukuba, Ibaraki Japan; 2 Research Center for Advanced Analysis, National Agriculture and Food Research Organization (NARO), Tsukuba, Ibaraki Japan; 3 Japan International Research Center for Agricultural Sciences, Tsukuba, Ibaraki Japan; 4 Graduate School of Life Sciences, Tohoku University, Sendai, Miyagi Japan

**Keywords:** Microbiology, Physiology

## Abstract

We investigated the potential dual application of two *Bradyrhizobium* strains (*B. diazoefficiens* USDA110 and *B. ottawaense* SG09) and plant growth-promoting bacteria, PGPB (*Pseudomonas* spp.: OFT2 and OFT5), to improve nodulation and N_2_-fixation in soybean plants. The growth-promoting effects of dual inoculation were observed on plant growth, physiology, and nodulation of soybean under normal conditions compared with plants individually inoculated with either USDA110 or SG09. Both OFT2 and OFT5 promoted N_2_-fixation by 11% and 56%, respectively, when dual inoculation with USDA110 and by 76% and 81%, respectively, when dual inoculation with SG09. Salinity stress significantly reduces soybean growth, physiology, nutrient uptake, nodulation, and N_2_-fixation. However, these adverse effects were attenuated by the dual inoculation of PGPB and rhizobia depending on the combination of inoculants. In particular, dual inoculation of PGPB with SG09 was more effective in enhancing the salt tolerance of soybean by reducing salt-induced ethylene production and improving nutrient uptake. However, no such effect was observed with the combined inoculation of USDA110 and OFT5. An effective symbiotic association between SG09 and two *Pseudomonas* bacteria can be considered a beneficial approach to improving the symbiotic efficiency of nodulation and mitigating salinity stress in soybeans.

## Introduction

Root nodule symbiosis between legumes and rhizobia leads to the fixation of atmospheric nitrogen into ammonia, the plant’s usable nitrogen form. This successful interaction resulted in the formation of N_2_-fixing nodules, by which the host plant can fix N_2_^1,2^. However, symbiotic interactions between rhizobia and their legume hosts are sensitive to environmental factors, including salinity^3^.

Soil salinity is a global environmental problem owing to ongoing climate change and is becoming a limiting factor for legume productivity^4^. Salinity indirectly affects root nodule symbiosis by reducing plant growth and available photosynthates or directly impacting nodule development and N_2_-fixation activity. Borucki and Sujkowska^5^ reported that moderate salt stress could negatively regulate several aspects of root nodule symbiosis, even if this concentration does not inhibit plant growth.

Plants respond to salinity stress conditions by increasing ethylene production, which is caused by the stress-induced accumulation of 1-aminocyclopropane-1-carboxylate (ACC), an ethylene precursor in higher plants. This phenomenon is referred to as ethylene stress. Ethylene has been shown to interfere with nodule formation by negatively affecting nodulation^6–8^. Bacteria producing ACC deaminase reduce ethylene biosynthesis by degrading ACC into α-ketobutyrate and ammonia^9^. Hence, these bacteria are referred to as ‘stress controllers’. Recently, bacterial ACC deaminase has received tremendous attention in root nodule symbiosis because it reduces ethylene levels in plant root tissues^10^. Several studies have reported that rhizobia expressing ACC deaminase can stimulate nodule formation and function, thus increasing the amount of fixed N_2_ under stress conditions^8,11–14^. However, it is important to note that not all rhizobia strains within a particular species produce sufficient amounts of the enzyme to degrade plant ACC^15^. Furthermore, Glick and Stearns^16^ reported that rhizobia typically exhibit only a low level of enzyme activity compared to free-living plant growth-promoting bacteria (PGPB) (i.e., 10- to 100-fold less than free-living bacteria). Thus, dual inoculation of non-ACC deaminase-producing rhizobia with high levels of ACC deaminase-producing PGPB should be a better solution for promoting biological N_2_-fixation in legumes.

In a previous study, two *Pseudomonas spp.* the OFT2 and OFT5 strains were isolated from the inner tissues of carrot and turnip crops, respectively, on organic farms in Ibaraki Prefecture, Tsukuba, Japan^17^. Both strains possess ACC deaminase and produce indole acetic acid (IAA) and IAA-like molecules that can promote plant growth in tomatoes, especially under NaCl stress conditions^18^. In preliminary experiments, both strains survived for 12 weeks in up to 137 mM NaCl in phosphate-buffered saline. The potential of ACC deaminase-producing PGPB to improve the symbiotic efficiency of co-inoculated rhizobia remains a significant challenge for proposing strategies to improve nodulation and N_2_-fixation in legumes under stress conditions. In this study, we investigated whether dual inoculation of rhizobia with ACC deaminase-producing *Pseudomonas* strains, OFT2 or OFT5, can synergistically facilitate plant growth and nodulation processes of two *Bradyrhizobium* strains (USDA110 and SG09) in soybean under normal and salinity stress conditions.

## Results

### Growth-promoting effects of PGPB by a single inoculation

**Figure 1:**
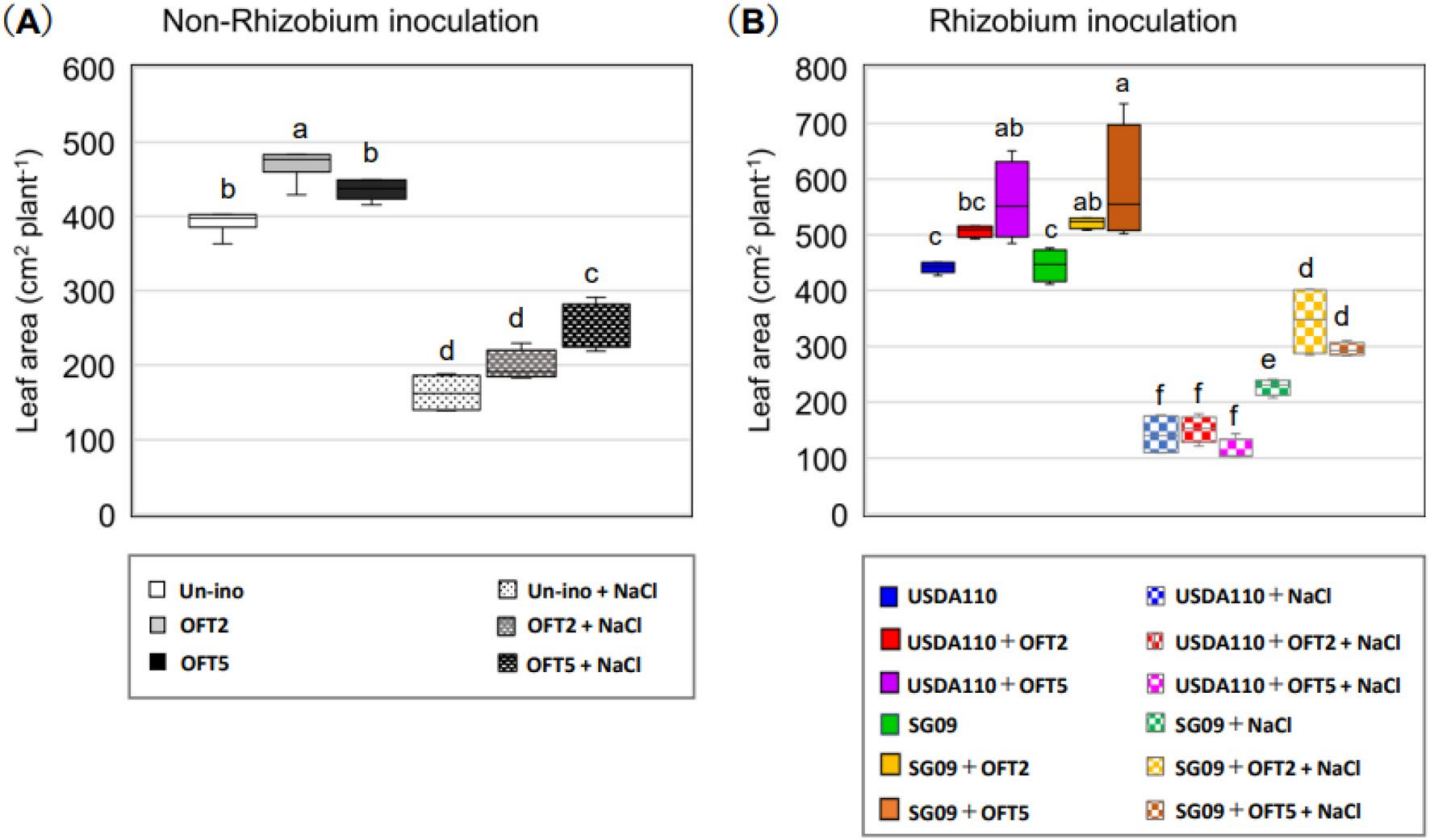
Effects of plant growth-promoting bacteria (PGPB) producing 1-amino-cyclopropane-1-carboxylate (ACC) deaminase on leaf area in rhizobia non-inoculated (**A**) and dual inoculated with rhizobial strains (**B**) in soybean plants grown under 0 and 60 mM NaCl. Different letters indicate classes that show significant differences (p < 0.05) using Duncan’s multiple range test (DMRT).

**Figure 2:**
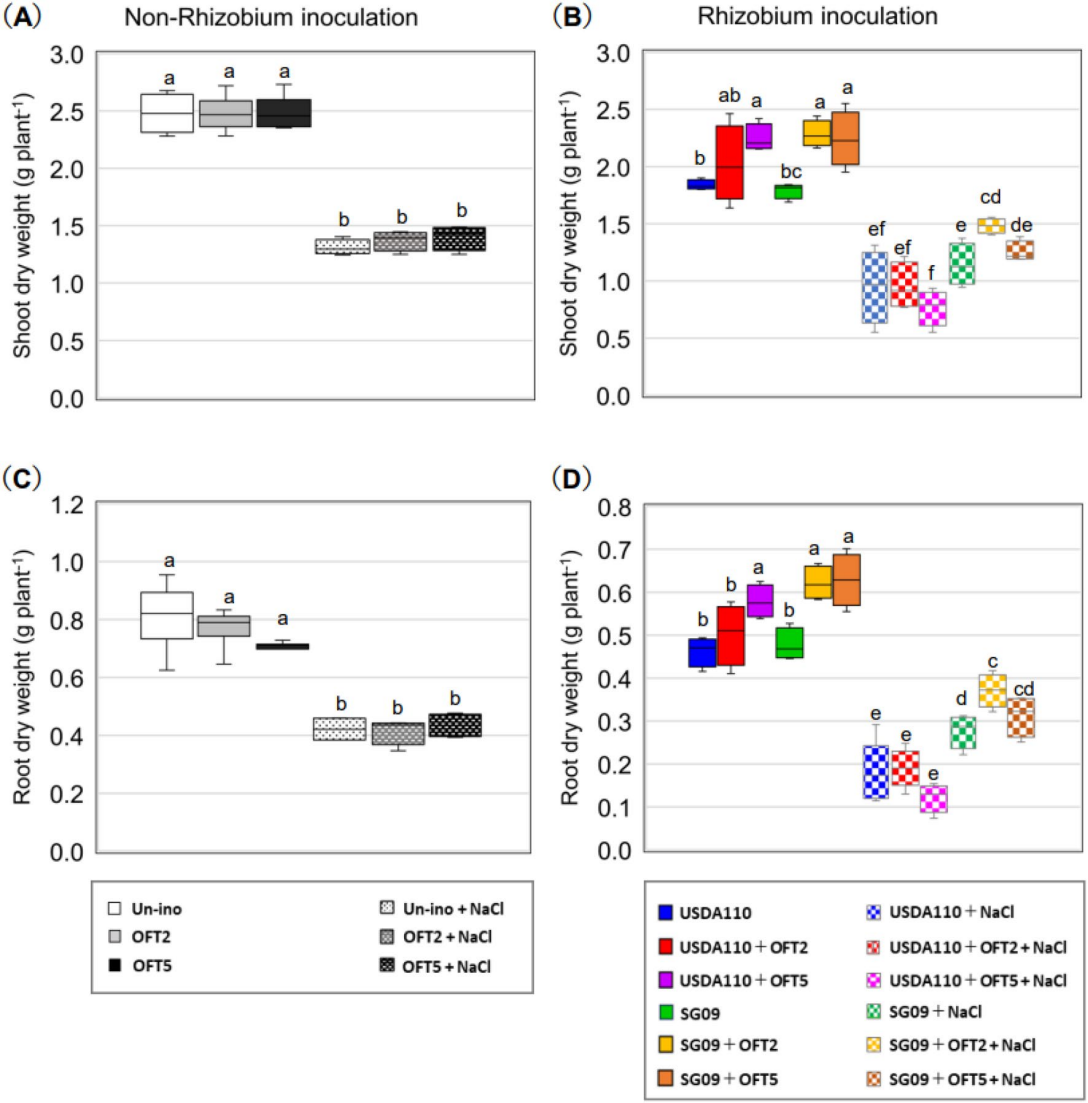
Effects of PGPB producing ACC deaminase on the shoot and root dry weight in rhizobia non-inoculated (**A,C**) and dual inoculated with rhizobial strains (**B,D**) in soybean plants grown under 0 and 60 mM NaCl. Different letters indicate classes that show significant differences (p < 0.05) using DMRT.

First, the growth-promoting effects of PGPB, OFT2, and OFT5, were examined without rhizobial inoculation (leaf area, shoot dry weight, and root dry weight; Figs. 1 A, 2A, C). Under 0 mM NaCl condition, no negative effects of PGPB were observed in all traits, whereas a growth promotion effect was detected only in leaf area with single inoculant OFT2 as compared with non-inoculated plants (Fig. 1A). A similar trend was observed under 60 mM NaCl conditions. Significant growth enhancement was only observed in leaf area with a single inoculant OFT5 compared to non-inoculated plants (Figs. 1A, 2A, C). These results indicate that the growth-promoting ability of OFT2 and OFT5 for soybean is not necessarily effective under either 0 mM or 60 mM NaCl conditions.

### Growth-promoting effects of PGPB by combined inoculation with rhizobia

First, the growth-promoting effects of rhizobia, USDA110, and SG09, were examined without inoculation with PGPB. No significant differences in leaf area, shoot dry weight, and root dry weight were observed between USDA110 and SG09 single-inoculated plants (Figs. 1B, 2B, D). PGPB inoculation increased leaf area when combined with USDA110 or SG09 under normal conditions. Consequently, USDA110 + OFT5 resulted in a significant increase in leaf area compared with USDA110. Furthermore, SG09 + OFT2 and SG09 + OFT5 significantly increased leaf area compared to SG09 (Fig. 1B). As for shoot growth, a significant increase in shoot dry weight was observed in USDA110 + OFT5 compared to USDA110 alone (Fig. 2B). Similarly, a significant increase in shoot dry weight was observed for SG09 + OFT2 and SG09 + OFT5, compared with SG09. The root dry weight of plants inoculated with either USDA110 or SG09 was significantly higher than that of their respective rhizobial single-inoculated plants (Fig. 2D). SG09 + OFT2 and SG09 + OFT5 had beneficial effects on root dry weight compared to the single inoculation of SG09.

### Growth response to salinity stress

Compared with normal conditions, salinity stress also severely restricted all growth traits of soybeans (Figs. 1, 2). The growth-promoting effect of PGPB in response to salinity stress varied depending on the combination of rhizobia and PGPB, especially the rhizobial strain. No significant difference was observed in the growth-promoting effect of PGPB in the combined inoculation of USDA110. However, a clear and significant increase in all growth traits was detected after dual inoculation with SG09. Plants with either SG09 + OFT2 or SG09 + OFT5 showed significant or numerical growth advantages compared to SG09. In particular, plants treated with SG09 + OFT2 showed significantly improved leaf area, shoot dry weight, and root dry weight under NaCl stress conditions than plants treated with SG09 alone (Figs. 1B, 2B, D). As a general trend, the single inoculant SG09 tended to exceed the single inoculant USDA110 in all growth traits.

### Salinity-induced leaf abscission

**Figure 3:**
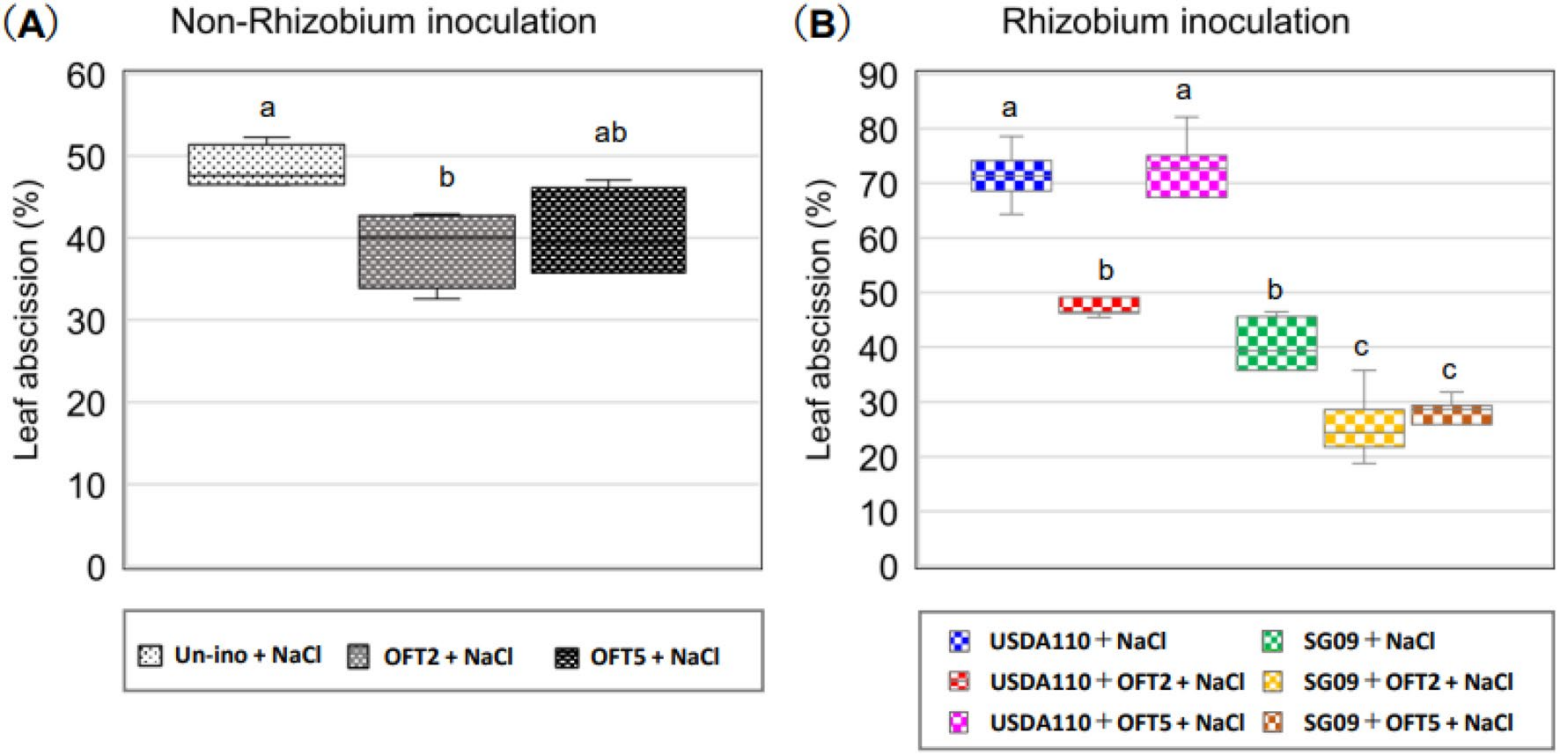
Effects of PGPB producing ACC deaminase on salinity-induced leaf abscission rate in rhizobia non-inoculated (**A**) and dual inoculated with rhizobial strains (**B**) in soybean plants grown under 60 mM NaCl. Different letters indicate classes that show significant differences (p < 0.05) using DMRT.

Salinity strongly induced leaf abscission in soybean regardless of rhizobial inoculation (Fig. 3, Supplementary Fig. S1 online). Among the rhizobial non-inoculated groups, OFT2 significantly reduced the leaf abscission rate compared to the non-inoculated group (Fig. 3A). Dual inoculation with USDA110 + OFT2 significantly reduced the leaf abscission rate compared to the single inoculant USDA110 (Fig. 3B). However, the USDA110 + OFT5 dual inoculant did not show such an effect. The dual inoculation effect was remarkable with SG09, and 36% and 34% reductions in leaf abscission rates were observed in SG09 + OFT2 and SG09 + OFT5, respectively, compared to SG09.

### Photosynthesis traits

The photosynthetic traits were significantly affected by inoculation with PGPB, rhizobial strains, and salinity stress (Table 1). The healthier plants grown under 0 mM NaCl had higher relative chlorophyll (SPAD values) and Phi2 and PhiNO ratios than more salinity-stressed plants but had a lower PhiNPQ ratio.

**Table 1:** Effects plant growth-promoting bacteria (PGPB) containing 1-amino-cyclopropane-1-carboxylate (ACC) deaminase on photosynthetic efficiency of photosystem II (Phi2), the yield of non-regulatory energy dissipation (PhiNO), the yield of non-photochemical quenching (PhiNPQ), and chlorophyll content (SPAD) in non-rhizobia inoculated and dual inoculated with rhizobial strains in soybean plants grown under 0 and 60 mM NaCl.

Traits		Phi2		PhiNO		PhiNPQ		SPAD	
Inoculation/treatments		0 mM	60 mM	0 mM	60 mM	0 mM	60 mM	0 mM	60 mM
Non-rhizobium	Un-inoculation	0.31 ± 0.03ab	0.20 ± 0.03c	0.14 ± 0.05a	0.13 ± 0.03ab	0.55 ± 0.05bc	0.67 ± 0.05a	27.8 ± 2.4a	18.4 ± 2.4b
	OFT2	0.32 ± 0.03a	0.25 ± 0.06bc	0.14 ± 0.02a	0.13 ± 0.04ab	0.55 ± 0.05bc	0.61 ± 0.05ab	28.1 ± 2.4a	24.4 ± 2.4a
	OFT5	0.39 ± 0.04a	0.33 ± 0.03a	0.17 ± 0.02a	0.15 ± 0.01a	0.44 ± 0.05c	0.49 ± 0.05c	27.6 ± 2.4a	23.5 ± 2.4ab
Rhizobium	USDA110	0.31 ± 0.04c	0.11 ± 0.04e	0.19 ± 0.04ab	0.05 ± 0.02f	0.50 ± 0.05c	0.84 ± 0.05a	29.1 ± 2.4b	17.3 ± 2.4f
	110 + OFT2	0.35 ± 0.03bc	0.23 ± 0.06d	0.19 ± 0.05ab	0.11 ± 0.01e	0.46 ± 0.05cd	0.68 ± 0.05b	29.4 ± 2.4b	21.3 ± 2.4de
	110 + OFT5	0.36 ± 0.01bc	0.24 ± 0.08d	0.18 ± 0.02abc	0.11 ± 0.04de	0.46 ± 0.05cd	0.65 ± 0.05b	29.5 ± 2.4b	19.1 ± 2.4ef
	SG09	0.35 ± 0.02bc	0.37 ± 0.06bc	0.16 ± 0.04abcd	0.13 ± 0.01cde	0.49 ± 0.05cd	0.50 ± 0.05c	29.3 ± 2.4b	23.8 ± 2.4cd
	SG09 + OFT2	0.40 ± 0.03ab	0.34 ± 0.03bc	0.21 ± 0.06a	0.14 ± 0.04bcde	0.39 ± 0.05d	0.53 ± 0.05c	32.9 ± 2.4a	25.1 ± 2.4c
	SG09 + OFT5	0.41 ± 0.04ab	0.44 ± 0.04a	0.19 ± 0.03ab	0.16 ± 0.02abcd	0.40 ± 0.05d	0.40 ± 0.05d	31.9 ± 2.4ab	24.8 ± 2.4c

Values are presented as means (S.D.), *n* = 4. Different letters indicate classes that show significant differences (p < 0.05) using Duncan’s multiple range test (DMRT).

The impact of the combined inoculation of PGPB and rhizobia on physiological traits was unclear under normal conditions. Under 60 mM NaCl conditions, a positive effect of PGPB was detected in both the USDA110 and SG09 inoculated groups. Dual inoculants USDA110 + OFT2 and USDA110 + OFT5 showed significantly higher Phi2 and lower PhiNPQ than USDA110. Similarly, SG09 + OFT5 had a higher Phi2 and lower PhiNPQ than SG09 alone. SG09 single-inoculated plants showed a relatively higher Phi2 than USDA110 single-inoculated plants under 0 mM NaCl conditions. Under 60 mM NaCl conditions, Phi2, PhiNO, and PhiNPQ significantly differed between SG09- and USDA110-inoculated plants.

Salinity also significantly decreased the SPAD values in plants. The increase in SPAD values due to OFT2 and OFT5 single inoculation tended to be inhibited by co-inoculation with USDA110. On the other hand, SG09 showed high SPAD values when inoculated alone and maintained this trend when co-inoculated with OFT2 and OFT5.

### Nodulation and N_2_-fixation of soybean

**Figure 4:**
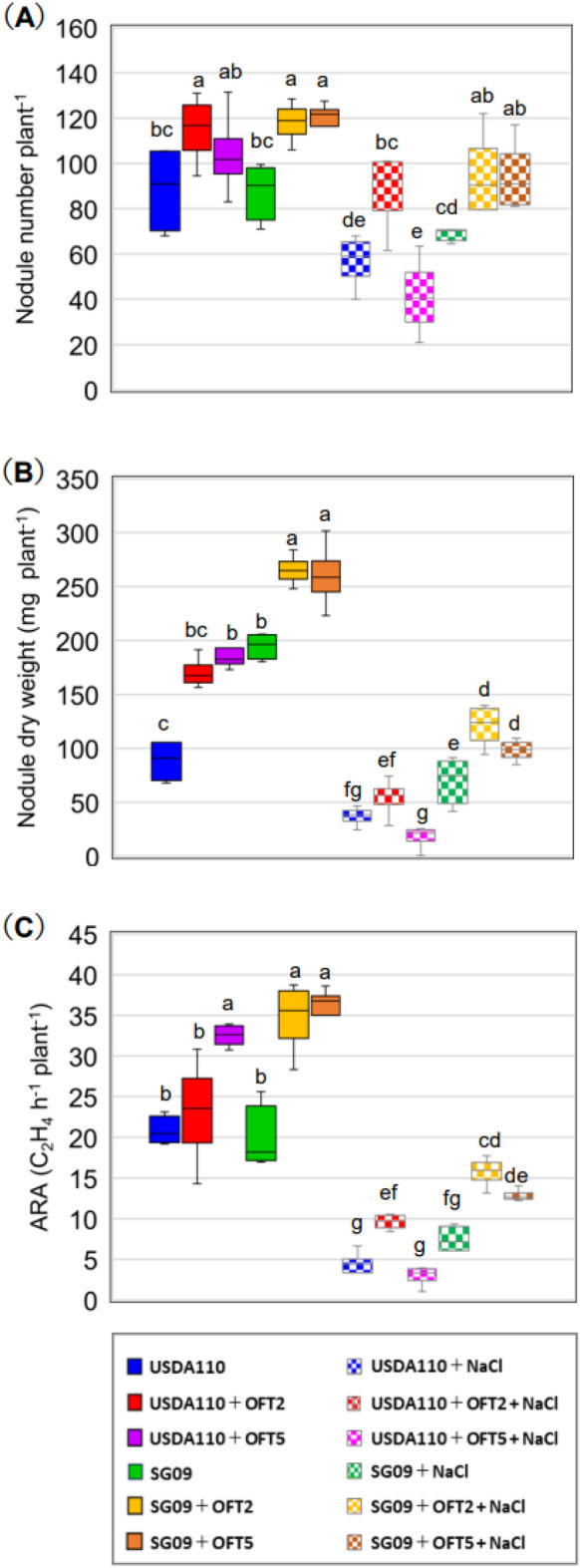
Dual inoculation effects of PGPB producing ACC deaminase on nodule number (**A**), nodule dry weight (**B**), and ARA (**C**) of soybean plants grown under 0 and 60 mM NaCl. Different letters indicate classes that show significant differences (p < 0.05) using DMRT.

The dual inoculant USDA110 + OFT2 resulted in a significantly greater nodule number by 29% compared with the single inoculant USDA110 (Fig. 4A). Furthermore, a significant increase in nodule number was achieved by 35% SG09 + OFT2 or SG09 + OFT5 compared with SG09. Similarly, nodule dry weight was significantly increased by dual inoculation with USDA110 + OFT5 by 24% compared to USDA110 (Fig. 4B). A similar trend was observed in the SG09-inoculation group: a significant increase in nodule dry weight by 36% with SG09 + OFT2 and 34% with SG09 + OFT5, compared to SG09. There was no significant difference in nodule number between USDA110 and SG09 (Fig. 4A). On the other hand, a significant difference in nodule dry weight between the two strains indicated that SG09-inoculated plants formed heavier nodules than plants inoculated with USDA110 (Fig. 4B). In the rhizobia-non-inoculated group, few nodules were formed on the host plants (less than 10) under normal conditions, and no nodulation was detected under 60 mM NaCl conditions (Supplementary Table S1 online).

Nodule number and dry weight were seriously limited in a saline environment. Salinity reduced nodule number by 36% in single inoculant USDA110 and 21% in single inoculant SG09, compared to their respective plants grown under normal conditions (Fig. 4A). The salinity-induced reduction in nodulation was mitigated by co-inoculation with PGPB. USDA110 + OFT2 showed significantly greater values for nodule dry weight than USDA110 alone (Fig. 4B). Among the SG09-inoculated groups, SG09 + OFT2 or SG09 + OFT5 had a considerable advantage in nodulation compared with the single inoculant SG09. A significant increase in nodule number was achieved by 35% of the plants treated with SG09 + OFT2 or SG09 + OFT5 compared with SG09 (Fig. 4A). The dual inoculation of SG09 + OFT2 benefited nodule dry weight by 39% and SG09 + OFT5 by 38% compared with SG09 alone (Fig. 4B). Salt damage affected the appearance of nodulated roots, and whitened ineffective nodules were observed (Supplementary Fig. S2D, J online). The combination of USDA110 + OFT2 suppressed the reduction of nodule number because of salinity compared to USDA110 alone (Fig. 4A). Furthermore, in the case of dual inoculation with SG09, SG09 + OFT2 and SG09 + OFT5 recovered both nodule number and dry weight compared to SG09 (Fig. 4A, B). No significant difference in nodule number was observed between USDA110 and SG09 inoculants (Fig. 4A). Similar to the normal condition, the nodule dry weight of the single inoculant SG09 was significantly higher than that of the single inoculant USDA110 (Fig. 4B).

The dual inoculation of rhizobia and PGPB revealed a large benefit in the N_2_-fixation (Fig. 4C). A significant enhancement of ARA was achieved by USDA110 + OFT5 relative to USDA110 under the no-salt condition. The greatest increase of more than 75% in ARA was confirmed in SG09 + OFT2 and SG09 + OFT5 plants, compared to SG09 alone. There was no significant difference in ARA between the plants inoculated with USDA110 and SG09. Under a single inoculation of rhizobia, N_2_- fixation in soybean plants was severely impaired by salt stress. N_2_-fixation efficiency in response to salinity stress depended on the rhizobia species and their combinations with PGPB. The dual inoculation of USDA110 + OFT2 gave about three times higher ARA than USDA110 alone. However, no such effect was observed for the USDA110 + OFT5. In contrast, the beneficial effects of N_2_-fixation under stress conditions were accompanied by dual inoculation of PGPB and SG09. SG09 + OFT2 and SG09 + OFT5 showed 112% and 74% higher ARA than SG09 alone. In the case of rhizobia, the single inoculant SG09 had a better ARA than that of USDA110.

### Ethylene production

**Figure 5:**
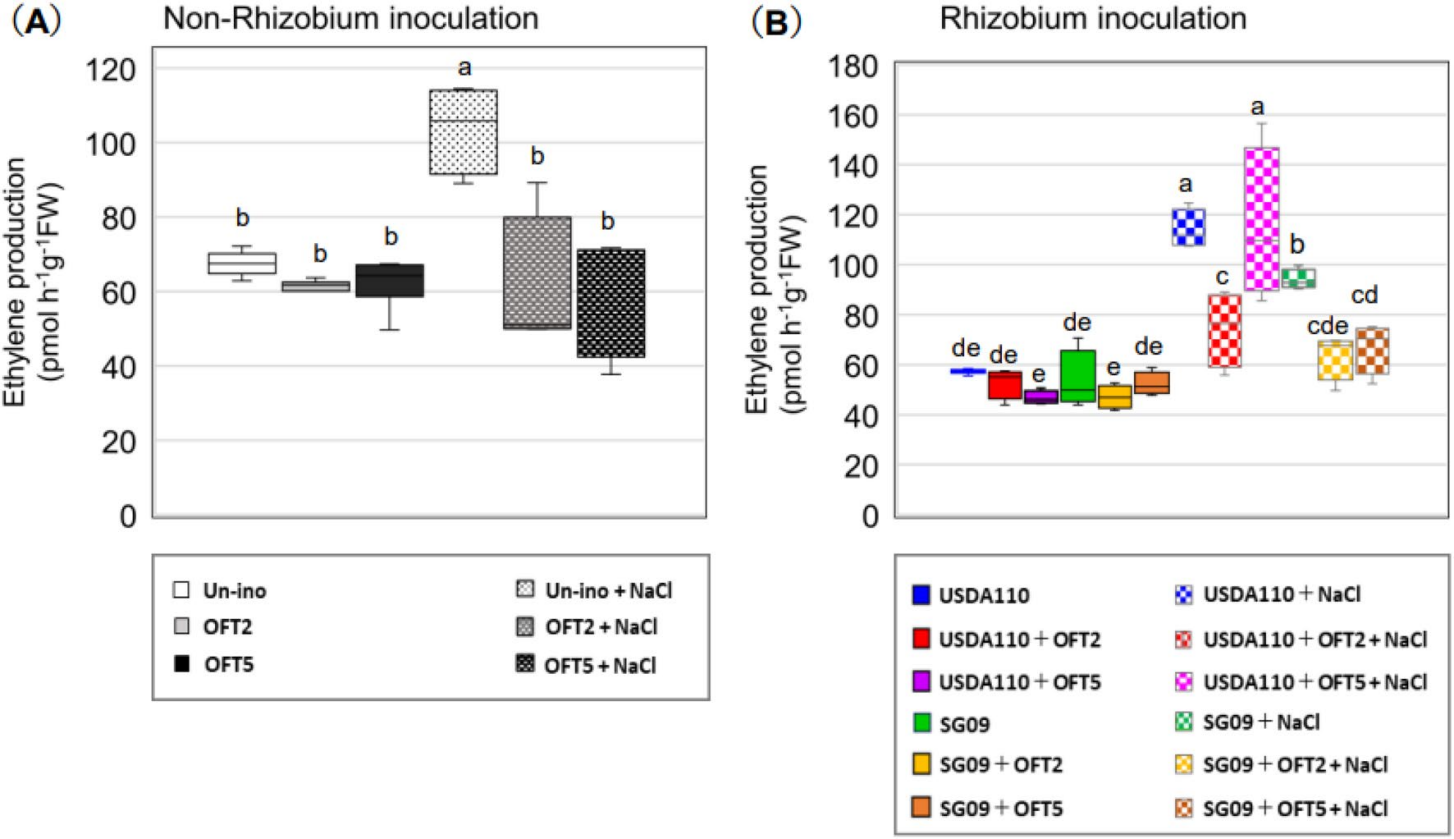
Effects of PGPB producing ACC deaminase on salinity-induced ethylene production in rhizobia non-inoculated (**A**) and dual inoculated with rhizobial strains (**B**) in soybean plants. Different letters indicate classes that show significant differences (p < 0.05) using DMRT.

Under no stress conditions, the seedlings inoculated with OFT2 or OFT5 showed slightly lower but no significant level of ethylene production compared to those without inoculation (Fig. 5A). NaCl treatment significantly increased ethylene production, while OFT2 and OFT5 inoculation suppressed the increase in ethylene production. Upon dual inoculation with rhizobia, the effect of PGPB was dependent on the combination of the rhizobial strains (Fig. 5B). Plants inoculated with USDA110 + OFT2 showed significantly reduced ethylene production, whereas those inoculated with USDA110 + OFT5 showed no or some reduction in ethylene levels. The effect of combined inoculation with SG09 and PGPB on reducing ethylene stress was more remarkable. Compared to single inoculation of SG09, dual inoculation of plants with SG09 + OFT2 and SG09 + OFT5 significantly reduced ethylene production.

### Metal-ion accumulation

The effect of PGPB on ion uptake in plants, with or without salinity, varied depending on the combination of rhizobia and PGPB (Table 2, Supplementary Table S2 online). Under normal conditions, shoot uptake (K, P, Mg, B) of SG09 + OFT2 and SG09 + OFT5 was higher than that of SG09 alone. Furthermore, the dual inoculant USDA110 + OFT5 showed higher K, Mg, Ca, and Mn uptake than the single inoculant USDA110. Under saline conditions, the specific rhizobial strain significantly influenced the accumulation of Na in the shoots. The lowest Na accumulation was observed in SG09, with or without PGPB, compared to USDA110 alone.

**Table 2:** Effects plant growth-promoting bacteria (PGPB) containing 1-amino-cyclopropane-1-carboxylate (ACC) deaminase on shoot ions uptake in non-rhizobia inoculated and dual inoculated with rhizobial strains in soybean plants grown under 0 and 60 mM NaCl.

Traits		Na		K		P	
Inoculation/treatments		0 mM	60 mM	0 mM	60 mM	0 mM	60 mM
Non-rhizobium	Un-inoculation	1.72 ± 0.79d	16.79 ± 3.81c	17.80 ± 1.41a	13.71 ± 1.17d	1.50 ± 0.14a	0.69 ± 0.09c
	OFT2	1.65 ± 0.43d	26.56 ± 3.59a	17.11 ± 1.34ab	14.52 ± 1.39cd	1.33 ± 0.07 b	0.70 ± 0.08c
	OFT5	1.75 ± 0.96d	21.02 ± 3.78 b	16.10 ± 0.57bc	15.90 ± 0.85bcd	1.31 ± 0.14 b	0.67 ± 0.11c
Rhizobium	USDA110	1.35 ± 0.17c	24.26 ± 14.25a	15.24 ± 1.36cde	11.40 ± 4.53fg	1.13 ± 0.04b	0.49 ± 0.20cd
	110 + OFT2	1.61 ± 0.23c	12.66 ± 5.28b	17.95 ± 3.96abc	12.41 ± 2.90ef	1.28 ± 0.21b	0.48 ± 0.07cd
	110 + OFT5	1.71 ± 0.55c	21.40 ± 3.5a	19.86 ± 2.53ab	16.61 ± 2.05g	1.31 ± 0.22b	0.37 ± 0.12d
	SG09	1.69 ± 0.93c	13.92 ± 1.38b	16.87 ± 0.78bcd	12.41 ± 1.51ef	1.10 ± 0.09b	0.55 ± 0.09cd
	SG09 + OFT2	1.28 ± 0.28c	12.12 ± 3.08b	20.44 ± 1.20ab	15.97 ± 1.12cde	1.24 ± 0.19b	0.66 ± 0.08c
	SG09 + OFT5	1.64 ± 0.24c	12.60 ± 2.12b	20.72 ± 2.75a	13.72 ± 1.06def	1.51 ± 0.23a	0.58 ± 0.09cd

Traits		Mg		Ca		Mn	
Inoculation/treatments		0 mM	60 mM	0 mM	60 mM	0 mM	60 mM
	Un-inoculation	3.09 ± 0.16a	2.19 ± 0.16b	1.08 ± 0.09a	0.76 ± 0.07b	9.42 ± 1.27a	6.14 ± 0.08c
	OFT2	3.19 ± 0.25a	2.21 ± 0.35b	1.08 ± 0.09a	0.77 ± 0.08b	8.13 ± 0.76ab	7.15 ± 1.23bc
	OFT5	2.97 ± 0.16a	2.09 ± 0.12b	1.04 ± 0.05a	0.78 ± 0.05b	6.75 ± 0.52bc	6.92 ± 0.84bc
	USDA110	2.48 ± 0.24cd	1.49 ± 0.41fg	0.89 ± 0.10bcd	0.54 ± 0.05ef	6.67 ± 1.00cdef	4.80 ± 2.00ef
	110 + OFT2	2.82 ± 0.61abc	1.48 ± 0.35fg	1.05 ± 0.20ab	0.58 ± 0.14ef	8.06 ± 1.48cd	5.74 ± 1.11def
	110 + OFT5	3.21 ± 0.33a	1.24 ± 0.23g	1.17 ± 0.14a	0.42 ± 0.09f	10.93 ± 2.33a	4.35 ± 1.08f
	SG09	2.61 ± 0.11bcd	1.60 ± 0.27fg	0.98 ± 0.07abc	0.64 ± 0.10e	8.46 ± 1.32bc	6.55 ± 1.45cdef
	SG09 + OFT2	3.25 ± 0.23a	2.15 ± 012de	1.16 ± 0.08a	0.85 ± 0.04cd	10.77 ± 2.11ab	8.09 ± 0.20cd
	SG09 + OFT5	3.25 ± 0.27ab	1.91 ± 0.30ef	1.12 ± 0.13a	0.71 ± 0.09de	8.54 ± 1.45bc	7.14 ± 1.36cde

Values are presented as means (S.D.), *n* = 4. Different letters indicate classes that show significant differences (p < 0.05) using Duncan’s multiple range test (DMRT).

### Root colonization of PGPB and rhizobial bacteria

**Figure 6:**
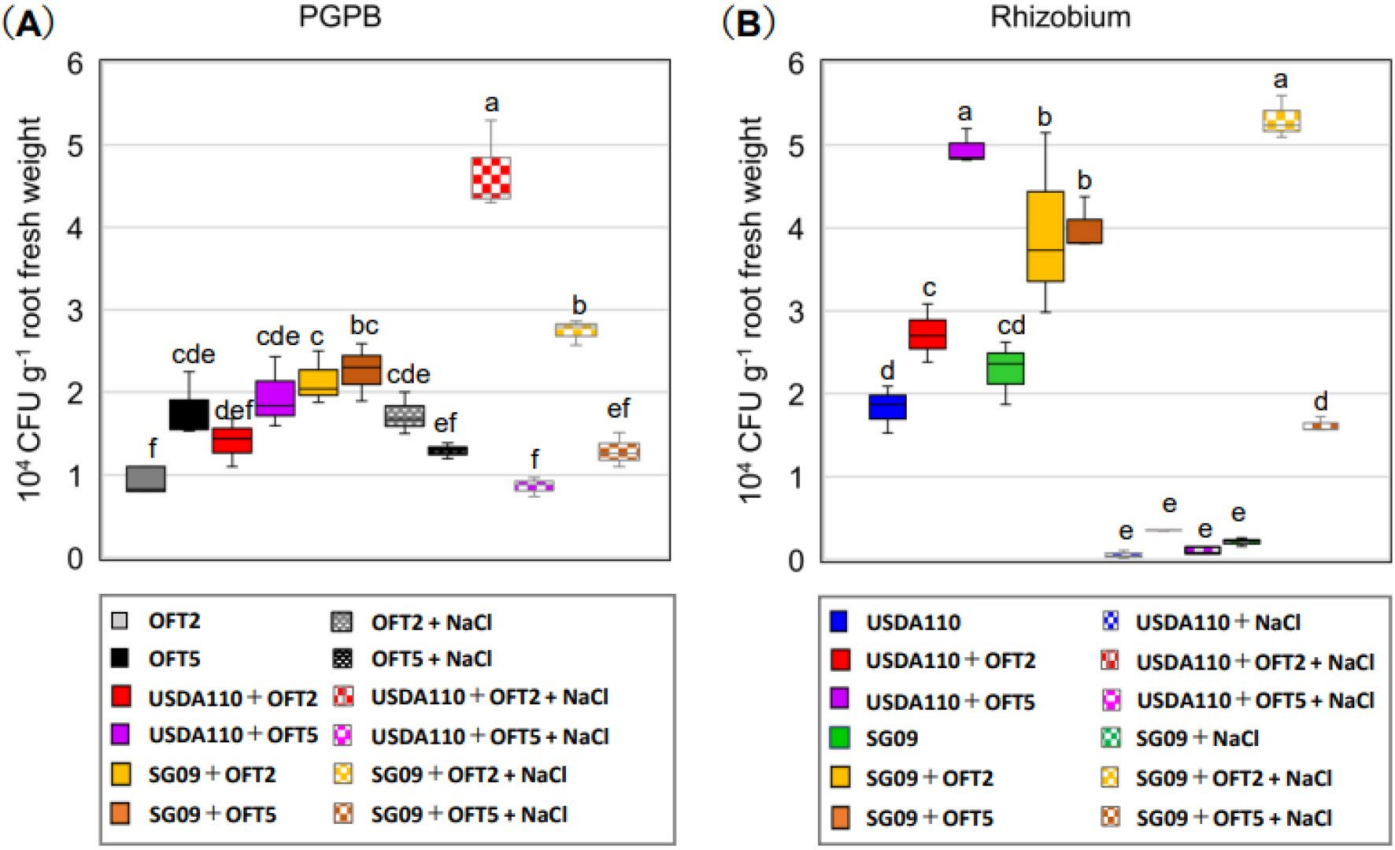
Colonization behavior of bacterial strains in the root of soybean plants grown under 0 and 60 mM NaCl for PGPB (**A**) and Rhizobium (**B**) over a 14-day in gnotobiotic vermiculite system. Different letters indicate classes that show significant differences (p < 0.05) using DMRT.

The colonization of *Pseudomonas spp* strains (OFT2 or OFT5) and *Bradyrhizobium* strains (USDA110 or SG09) in the roots of soybean cultivated under non-saline and saline conditions were assessed by determining the colony-forming units (CFU) per gram of root fresh weight (Fig. 6). In the non-saline condition, the population density of OFT2 and OFT5 cells in the fresh root of 14-day-old seedlings was measured at 0.97 × 10^4^ and 1.78 × 10^4^ CFU g^−1^ fresh root, respectively. The density of soybean plants inoculated with either *Bradyrhizobium* strain USDA110 or SG09 exhibited a remarkable similarity. Notably, the population counts for strains USDA110 and SG09 were measured at 1.83 × 10^4^ CFU g^−1^ and 2.28 × 10^4^ CFU g^−1^, respectively.

The cell density of OFT5 did not increase when co-inoculated with USDA110 or SG09 (Fig. 6A). However, co-inoculation of PGPB (OFT2 or OFT5) improved the colonization of *Bradyrhizobium strains* (USDA110 or SG09; Fig. 5B). The rhizobial cell population increased two to threefold in USDA110 and twofold in SG09 when co-inoculated with OFT5 or OFT2, respectively.

Salinity significantly decreased the colonization of *Bradyrhizobium* strains (USDA110 or SG09) when singly inoculated (Fig. 6B). However, no negative effect of salinity was observed for both OFT2 and OFT5 (Fig. 6A). Salinity significantly reduced the colonization of OFT5 when co-inoculated with USDA110 or SG09 compared to non-saline conditions. On the other hand, the presence of 60 mM of NaCl significantly increased the colonization of OFT2 with either USDA110 or SG09. Salinity-induced reduction in the colonization of USDA110 was observed when co-inoculated with either OFT2 or OFT5 (Fig. 6B). However, the presence of OFT2 and OFT5 in the soybean root rescued the population of SG09 when compared with sole SG09 inoculation.

### Dual inoculation efficacy between SG09 and PGPB in response to salinity

For SG09 and PGPB, which showed the highest dual inoculation efficacy, each index value was validated under salt stress conditions in an independent experiment. Multiple comparisons between inoculation conditions were performed. Similar results were obtained in the re-experiment, where dual inoculation of either SG09 + OFT2 or SG09 + OFT5 under salinity stress showed a significant improvement in the adverse effects of salinity stress on plant growth, physiological traits, nodulation, and N_2_-fixation (Table 3). In response to salinity stress, the reduction in growth and nodule number in the single inoculant SG09 was remarkable and was two times lower than those of SG09 + OFT2 or SG09 + OFT5.

**Table 3:** Effect of plant growth-promoting bacteria (PGPB) expressing 1-amino-cyclopropane-1-carboxylate (ACC) deaminase on recovery of soybean plants in terms of growth, physiological traits, and nodulation of soybean grown under salinity for 11 days conditions.

	Shoot dry weight (g)	Root dry weight (g)	Chlorophyll content (SPAD)	Phi2	PhiNo	PhiNPQ	Nodule No	Nodule DW (mg)	ARA/plant (nL C_2_H_4_ h^−1^ plant^−1^)
SG09	35.96 ± 6.5a	2.95 ± 0.7a	48.00 ± 0.4a	0.54 ± 0.02a	0.23 ± 0.01a	0.23 ± 0.03b	372.25 ± 36.5a	816.65 ± 166.3a	24.76 ± 7.5a
SG09 + NaCl	7.54 ± 1.6c	1.16 ± 0.2b	29.55 ± 5.2c	0.22 ± 0.03c	0.13 ± 0.04c	0.59 ± 0.09a	152.81 ± 42.5b	222.50 ± 39.8c	12.40 ± 1.6c
SG09 + OFT2 + NaCl	15.16 ± 3.9b	1.73 ± 0.3b	38.88 ± 3.7b	0.3 ± 0.03b	0.17 ± 0.02bc	0.52 ± 0.04a	304.88 ± 38.7a	409.38 ± 121.1b	14.45 ± 2.6bc
SG09 + OFT5 + NaCl	17.28 ± 4.7b	1.73 ± 0.2b	41.93 ± 2.1b	0.30 ± 0.03b	0.18 ± 0.03ab	0.51 ± 0.03a	318.50 ± 33.2a	452.25 ± 84.3b	18.56 ± 3.0b

Values are presented as means (S.D.), *n* = 4. Different letters indicate classes that show significant differences (p < 0.05) using Duncan’s multiple range test (DMRT) (p < 0.05).

## Discussions

### PGPB effects on plant growth

The effects of ACC deaminase-producing bacteria on plant growth under normal and stress conditions are well known^9,18^. However, the detailed mechanisms influencing root nodule symbiosis remain unclear^10^. In this study, we investigated the single inoculation of the two rhizobial strains (USDA110 and SG09) and their dual inoculation with the two *Pseudomonas* strains (OFT2 or OFT5) on the growth and N_2_-fixation activity of soybean under normal and salinity stress conditions. Our results demonstrated that the dual inoculation of PGPB enhanced plant growth, nutrient uptake, and photosynthetic traits with rhizobia.

Under normal conditions, OFT2 and OFT5 exhibited growth-promoting effects when co-inoculated with USDA110 and SG09 (Figs. 1, 2, 3, Supplemental Fig. 1). In general, the production of phytohormones, especially IAA, by PGPB has a positive impact on host plant development, particularly in the root system^25,26^. Therefore, co-inoculation of legumes with rhizobia and PGPB is believed to enhance rhizobial symbiotic capacity by increasing the amount and number of roots available for nodule formation^27,28^. However, when inoculated alone on soybean and tomato, OFT2 and OFT5 did not significantly increase host plant root biomass (Fig. 2C, Win et al.^18^). On the other hand, co-inoculation of PGPB with rhizobia increased soybean biomass (Fig. 2D). This suggests that the actions of dual-inoculated bacteria on soybean roots may produce complementary growth-promoting effects.

During the initial exposure to salinity, plants undergo water stress, which reduces leaf area by preventing water loss due to osmotic pressure^29^. The restoration of leaf area reduction was highly dependent on the combination with rhizobial strains. During prolonged exposure to salinity, ionic stress leads to the death of mature and a reduction in photosynthetic leaf area^30^, decreasing plant biomass. Inoculation with both OFT2 and OFT5 improved the leaf area rate of leaf ablation. However, it is highly dependent on the strain of the co-inoculated rhizobia. The leaf abscission rate was significantly reduced with USDA110 + OFT2 compared to USDA110 plants or with SG09 + OFT2 and SG09 + OFT5 compared to SG09.

The reduction in leaf area due to osmotic stress^29^, the leaf abscission rate due to ionic toxicity from salinity stress^31^, and the decrease in chlorophyll content are largely attributed to the reduction in photosynthetic activity in legumes^32^. The quantum yields of photosystem II (Phi2), unregulated processes (PhiNO), and non-photochemical quenching (PhiNPQ) were significantly influenced by salinity stress (Table 1). These photosynthetic traits were improved by PGPB inoculation, leading to an increase in Phi2 and a decrease in PhiNPQ in soybean plants, depending on the treatment combination.

Notably, under salt stress conditions, significant inhibition of Na^+^ absorption was observed in USDA110 + OFT2, SG09 + OFT2, and SG09 + OFT5, where growth-promoting effects were observed (Table 2). These effects may provide plants with balanced macro- and micronutrient nutrition^33^. In the dual inoculation treatments, plants exhibited significantly or numerically higher shoot content of K, Ca, and Mg compared to their respective plants in the presence or absence of NaCl stress. Plant-associated PGPB enhances nutrient uptake and accumulates through various mechanisms^34^. High levels of K, Ca, and Mg accumulation in plants can also facilitate the maintenance of high ion/Na ratios in saline soil, thereby mitigating the adverse effects of Na on plant growth^35^. Furthermore, PGPB-inoculated plants showed elevated shoot uptake of P, Mg, and Ca both in the presence and absence of salt stress, particularly when combined with specific rhizobia (Table 2).

### PGPB effects on nodule formation and nitrogen fixation

Our study revealed that dual inoculation with PGPB and rhizobia increased the number of nodules, nodule dry weight, and ARA under salt-free conditions (Fig. 4). A meta-analysis by Zeffa et al. on the effects of *Bradyrhizobium* and PGPB co-inoculation on soybean root nodule symbiosis supported our findings, as the analysis of 42 papers demonstrated significant increases in nodule number, nodule weight, root biomass, and shoot biomass^36^. The colonization of rhizobia on the root surface plays a crucial role in nodule formation^37^. The four strains used in this study exhibited distinct behaviors depending on the presence or absence of salt stress and the combination of co-inoculated bacteria (Fig. 6). Regarding bacterial colonization, OFT5 showed a strong tendency for consistent colony formation levels, regardless of the rhizobial strain and the presence or absence of salt stress, indicating low sensitivity to both biotic and abiotic environmental factors. In contrast, OFT2 exhibited accelerated colony formation levels when co-inoculated with SG09 and USDA110, suggesting a high sensitivity to the external biological environment and an ability to adapt its growth in response to co-inoculated rhizobia.

When considering the rhizobial strains, colony formation levels of USDA110 were enhanced depending on the presence of OFT2 and OFT5, but this promotional effect diminished under salt stress conditions. Supplementary Table S3 (online) indicates that USDA110 exhibited lower salt stress tolerance compared to SG09. Therefore, the observed change in colony formation levels of USDA110 under salt stress conditions is likely influenced, at least partially, by the salt stress itself. In contrast, SG09 exhibited increased colony formation levels in both the presence and absence of salt stress, possibly owing to its higher salt stress tolerance compared to USDA110.

As observed in previous studies^5,38,39^, the symbiotic interactions between soybean plants and rhizobia are strongly influenced by salinity, resulting in a reduction in nodulation and N_2_-fixation (Fig. 4). PGPB inoculation restored nodulation and N_2_-fixation; with OFT2 and OFT5 having the greatest effect on SG09, whereas OFT5 had no significant effect on USDA110. Soil salinity reduces the survival and proliferation of rhizobia in the soil and rhizosphere of leguminous plants^40^. Similarly, we observed a significant decrease in rhizobial colonization in soybean roots under salinity stress (Fig. 6B). This decrease in colony formation frequency may result in a reduction in potential rhizobial infection sites and the number of nodules formed^41^. The reduction in rhizobial colonization was restored by the application of PGPB. Specifically, the presence of OFT2 or OFT5 promoted colonization by SG09, while no such promotion was observed by USDA110 (Fig. 6B). It is worth noting that the interaction with PGPB differs between SG09 and USDA110. A notable result observed under salinity conditions is the inhibitory interaction between USDA110 and OFT5, particularly in terms of nodulation phenotype and plant growth. Salinity can adversely affect the population size of USDA110 cells and their ability to colonize soybean roots and form nodules (Supplementary Table S3 and Fig. 6B). This can lead to a decrease in N_2_-fixation and subsequent nitrogen starvation in plants when using a nitrogen-free nutrient application. Limited nitrogen availability may promote competition between microorganisms (PGPB) and the host plant for nutrients, resulting in decreased growth and reduced symbiosis efficiency of the host plant^42^.

Soil microorganisms do not exist alone but are members of the soil microflora, and the networks among microorganisms are known to be crucial for the stability of soil microflora^43,44^. Although our study used a limited experimental system of co-inoculation with two microorganisms, further studies are warranted to determine the differential effects of coexisting PGPB on the symbiotic potential of microorganisms in host plants.

### PGPB effects on ethylene production

In plants exposed to salt, changes in root morphology are induced as a common adaptation mechanism, and stress-induced ethylene hormones play a key role in this process^18^. Furthermore, ethylene can inhibit various steps of the rhizobia-initiated nodulation process in legumes by inhibiting nodule formation and function^10,15,45–47^. Our results indicated that OFT2 and OFT5 significantly reduced stress-induced ethylene levels compared to the non-inoculated control (Fig. 5A). Similarly, findings have been reported, where dual inoculation of rhizobia with ACC deaminase-producing bacteria enhances nodulation and N_2_- fixation by reducing ethylene synthesis^10,38,48,49^.

Stress-related ethylene production depended on combinations of PGPB and rhizobial strains. Specifically, OFT5 bacteria did not affect stress-related ethylene emissions in soybean plants inoculated with USDA110, whereas OFT2-inoculated plants showed reduced ethylene emissions in all the treatment groups (Fig. 5B). The results suggest that OFT2 and OFT5 employ different strategies to alleviate salt stress depending on the rhizobial strains.

### *Bradyrhizobium ottawaense* SG09, a potential rhizobial inoculum for agriculture

Symbiotic N_2_-fixation is dependent on the host plant genotype, rhizobial strains, and the interaction of these symbionts with pedoclimatic factors and environmental conditions^50^. When comparing rhizobial inocula alone, no significant difference was observed between USDA110 and SG09 under normal conditions. However, SG09 exhibited higher growth, physiology, and nodulation capacities under salt conditions (Figs. 1, 2, 4, Table 1). This may be related to the lower ethylene synthesis in SG09-inoculated plants compared to USDA110 alone (Fig. 5B). Genomic data analysis revealed that both USDA110 and SG09 encode an ACC deaminase gene (acdS, Locus tag = ‘blr0241’ and ‘SG09_14070’, respectively). Although the expression level of the ACCD gene in the two rhizobia and their ability to degrade ACC are unknown, our results may imply the possibility that SG09 is a rhizobacterium with PGPB-like abilities. This opens up the possibility for further studies that should consider the gene expression levels of different rhizobia under salt stress.

In this paper, we analyzed soybean plants after 5 weeks of inoculation with PGPB and rhizobia and revealed the promoting effect of PGPB on root nodule symbiosis. Several reports have demonstrated the effectiveness of PGPB in promoting root nodule symbiosis, while no significant promoting effect on soybean yield has been clearly demonstrated^36^. Therefore, verification of the PGPB effect until soybean harvest is necessary for the functional use of PGPB in agriculture. Our study also suggests that identifying combinations of PGPB and rhizobacteria that have a promotive effect on plant growth and root nodule symbiosis is important for the development of more efficient soybean inoculants. In our system, co-inoculation of SG09 with OFT2 or OFT5 may be the most effective, considering tolerance to salt stress. *Bradyrhizobium ottawaense* SG09 is a rhizobium that possesses the *nosZ* gene encoding nitrous oxide reductase, which can reduce N_2_O released from the soil^19^. Evaluation of whether the co-inoculation of PGPB and SG09 can increase soybean yield and reduce N_2_O gas emissions in agricultural fields is a future research goal for us.

## Conclusion

In general, these results for promoting growth, nodulation, and N_2_-fixation indicate that under stress-free conditions, the combined inoculation of USDA110 + OFT5, SG09 + OFT2, and SG09 + OFT5 is the best way to improve nodulation and N_2_-fixation in soybean plants. However, under salinity stress conditions, less damage to the nodulation potential of soybean was only achieved with the combined use of SG09 with either OFT2 or OFT5. Compared with SG09, USDA110 inoculation in the Enrei cultivar is not a good choice for growing in salinity-affected areas in terms of nodulation and N_2_-fixation. However, dual inoculation with USDA110 and OFT2 mitigated salinity-induced damage in nodulation and N_2_-fixation. Our study showed that dual inoculation of SG09 with either OFT2 or OFT5 significantly improved plant growth, shoot, and root biomass, as well as nodulation and N_2_-fixation activity under salt stress. Dual inoculation between ACC deaminase-producing *Pseudomonas spp* and *Bradyrhizobium* can be used as a biofertilizer to synergistically promote nodulation and N_2_-fixation in soybean in normal and saline soils. However, further research is needed to evaluate the efficacy of these strains in different soybean genotypes, as well as under actual field conditions and at multiple locations, to confirm the promoting effects of PGPB.

## Materials and methods

### Rhizobium strains and growth conditions

Two *Bradyrhizobium* strains, *B. diazoefficiens* USDA110 and *B. ottawaesense* SG09, were used in the present study. *B. ottawaesense* SG09 was previously isolated from sorghum (*Sorghum bicolor*) grown in Fukushima, Japan. SG09 strain possesses *nif* and *nod* genes for N_2_-fixation and nodulation and *nos* genes that catalyze N_2_O reduction to N_2_^19^. For inoculum preparation, *B. diazoefficiens* USDA110 and *B. ottawaesense* SG09 were grown in tryptone yeast (TY broth) and HM (HM broth) media, respectively, at 28 °C and 120 rpm for 96 h. This inoculum was used for the inoculation. The composition of TY broth (grams per liter) was 5.0 of Tryptone, 3.0 of yeast extract, and 0.9 of CaCl_2_. 2H_2_O. A HM broth consisted of (grams per liter): HEPES, 0.10; MES, 0.10; MgSO_4_.7H_2_0, 0.18; FeCls, 0.004; CaCl_2_.2H_2_0, 0.013; NaSO_4_, 0.25; NH_4_Cl, 0.32; supplemented with 1 g l-Arabinose and 0.25 g yeast extract (Difco, Detroit, MI).

### Plant material and growth conditions

Seeds of the soybean cultivar ‘Enrei’ were sterilized by fumigation with sodium hypochlorite and concentrated hydrochloric acid solutions for 24 h. Enrei (GmJMC025) seeds were acquired from Genebank Project NARO (https://www.gene.affrc.go.jp/databases-core_collections_jg.php). The seeds were then pre-germinated in sterilized and water-moistened vermiculite at 28 °C for 1 day. Two uniform one-day pre-germinated seeds with 0.5 cm root radicals were planted in plastic pots (12 cm × 12 cm × 20 cm; 1,000 mL) filled with autoclaved vermiculite and nutrient-free granular soil (Kanuma Sangyo Co., Tochigi, Japan) (1:1). The pots were inoculated with 5 × 10^6^ cells mL^−1^ of *Bradyrhizobium* strains (USDA110 or SG09) thoroughly mixed with 500 mL of nitrogen-free (Broughton and Dilworth) B & D nutrient solution^20^ containing 0 or 60 mM NaCl. The pots were then grown in a greenhouse with controlled environmental conditions at the premises of the National Agriculture and food research organization (NARO, Japan). Greenhouse conditions were temperatures typically at 25 °C/20 °C (day/night) under natural light conditions.

Two plant growth-promoting bacteria, *Pseudomonas* spp. strains OFT2 and OFT5 were chosen based on the reports of Win et al.^18,21,22^. They were grown in tryptic soy broth at 28 °C and 150 rpm for 24 h. Five to six days after the emergence of the seedlings, 1 mL (approximately 10^7^ cells mL^−1^) of the bacterial suspension was poured concentrically around the roots of each pot. The pots were arranged in a completely randomized design with four replicates in a greenhouse. Pot positions were randomly changed daily to minimize positional effects, and the plant density and size were small enough to induce mutual shading among different plants.

After seed sowing and bacterial inoculation, the pots were irrigated with 500 mL of nitrogen-free B & D nutrient solution with 0 or 60 mM NaCl three times a week for 21 days for the plants in the rhizobia-inoculated group (USDA110 alone, SG09 alone, and USDA110/SG09 with OFT2/OFT5), no nitrogen was applied because N_2_-fixation provides nitrogen to host plants. On the other hand, when plants were grown under nitrogen-free, 60 mM NaCl conditions, rhizobial non-inoculated plants could not grow adequately. Thus, plants belonging to the rhizobia non-inoculated group (Un-inoculation, OFT2 alone, and OFT5 alone) were watered with the same B & D nutrient solution supplemented with 0.5 mM of NH_4_NO_3_ to prevent plant withering. Three weeks after salinity stress, the pots were irrigated with salt-free B & D nutrient solutions until harvest. All plants were harvested 5 weeks after sowing. Total leaf area, shoot and root dry weight, nodule number, nodule dry weight, and N_2_-fixation activity (ARA) were recorded. These measurements were expressed as units per plant.

### Chlorophyll contents, photosynthesis traits

The photosynthetic efficiency of photosystem II (Phi2), yield of non-regulatory energy dissipation (PhiNO), yield of non-photochemical quenching (PhiNPQ), and leaf chlorophyll content (SPAD) were measured on the second fully open leaf from the top of the plants using MultispeQ (PhotosynQ, East Lansing, MI, USA) (n = 8). Measurements were performed 3 weeks after salinity stress on a sunny day from 9:00 am to 11:00 am.

### N fixation measurement

Upon harvest, N_2_-fixation activity was measured using an acetylene reduction assay (ARA)^23^. Briefly, the entire root system with nodules of soybean plants was incubated in a 100 mL sealed glass vial, from which 10% (v/v) air was replaced with pure acetylene. The samples were incubated at 25 °C for 20 min to convert acetylene into ethylene. One milliliter of headspace was injected into a gas chromatograph (GC-2014 SHIMADZU, Kyoto, Japan) equipped with a flame ionization detector (FID) and Porapak-N to measure ethylene gas. There were four replicates per treatment, and one replicate represented two plants (n = 8). Nitrogen fixation was expressed as nL C_2_H_4_ plant^-1^ h^–1^ compared with the standard curve of pure ethylene.

### Measurements of growth parameters

At harvest, the aboveground parts of plants were separated into stems and leaves. Each leaf was photographed, and the total leaf area (LA) was calculated using ImageJ software. The leaves, shoots, and roots were oven-dried at 70 °C for 72 h, and the dry weights were determined. These measurements were expressed as units per plant. The leaf abscission rate of plants was measured by counting the number of dead leaves at harvest.

### Monitoring of ethylene production by seedlings

Ethylene emission from soybean seedlings of each treatment was measured according to the protocol described by Win et al.^18^, with slight modifications. Briefly, four uniform one-day pre-germinated seeds were sown in 300-mL plant box (CUL-JAR300; Iwaki, Tokyo, Japan) filled with a mixture of autoclaved vermiculite and nutrient-free granular soil (1:1 ratio). The plants were then grown in a temperature-controlled growth chamber. The plant growth condition and bacteria inoculation followed the previously described methods.

After five days of sprouting, the seedlings were irrigated with B & D nutrient solution mixed with 200 mM NaCl for a duration of 12 d. Similarly, for plants in the rhizobia non-inoculated group (including un-inoculation, OFT2 alone, and OFT5 alone), the same amount of B & D nutrient solution (0 mM or 200 mM NaCl) supplemented with 0.5 mM of NH_4_NO_3_ was used for watering, in order to prevent plant withering. Subsequently, soybean seedlings were uprooted, and their root zones were gently washed to avoid damaging the root systems. The uprooted seedlings were immediately placed inside 100 mL glass vials. The vials were opened for 30 min to allow the air to escape and then sealed with rubber septa for 24 h. After this period, air samples from the vial headspace were collected. One milliliter of the headspace sample was injected into a gas chromatograph (GC-2014 SHIMADZU, Kyoto, Japan) equipped with a FID. The amount of ethylene evolved was quantified as picomoles of ethylene per gram of fresh weight, using a standard curve of pure ethylene (n = 4 for each treatment).

### Mineral-ion analysis

For quantification of ion content in the shoots, 20 mg of dried sample (fine powder) was digested in 300 μl of concentrated nitric acid (HNO_3_), incubated at 95 °C for 3–4 h, cooled at room temperature for 1 h, and the volume was made up to 10 mL with Milli-Q water^18,24^. Mineral ion analysis of plant extracts was performed using inductively coupled plasma mass spectrometry (Agilent 7700X, USA).

### Isolation of introduced bacteria from soybean roots

Soybean roots were co-inoculated with *Pseudomonas spp*. strains (OFT2 or OFT5) and *Bradyrhizobium* strains (USDA110 or SG09) to investigate colony formation under sterile conditions using an autoclave inoculation-plant box. Salinity conditions were established by adding 0 and 60 mM NaCl to the B & D nutrient solution. Treatments without rhizobial inoculation utilized B & D nutrient solution supplemented with 0.5 mM NH_4_NO_3_. For the inoculation, the pots were treated with 5 × 10^6^ cells mL^−1^ of *Bradyrhizobium* strains (USDA110 or SG09) thoroughly mixed with 200 mL of nitrogen-free B & D nutrient solution containing 0 and 60 mM NaCl. After seed sowing and rhizobium inoculation, the plant box was covered with an inverted-300 mL transparent plastic cup (12 cm height) with three holes sealed with Millipore membrane to provide partial aeration. and the cup was further sealed with parafilm. Four days after the seedling emerged, 1 mL (approximately 10^7^ cells mL^−1^) of the bacterial suspension (OFT2 or OFT5) was poured concentrically around the roots. The seedlings were then cultivated for 14 days in a growth cabinet under a 16-h light period at 25 °C and an 8-h dark period at 16 °C.

During sampling, the roots were gently washed in autoclaved milli-Q water to remove vermiculite. The entire roots were surface sterilized by immersing them in 1.0% sodium hypochlorite for 30 s and then washed five times in autoclaved milli-Q water. Three whole roots per treatment were crushed together using a pestle and mortar. One gram of crushed root sample was mixed with 10 mL of phosphate buffer. The homogenates were serially diluted and spread on two agar plates. TSA medium supplemented with 100 mg L^−1^ of streptomycin and rifampicin was used for the selection of *Pseudomonas* strain (OFT2, OFT5). *Bradyrhizobium* strains (USDA110 or SG09) were selected on TY or HM agar supplemented with 50 mg L^−1^ polymyxin B sulfate (Sigma-ALDRICH), respectively. The number of bacterial cells colonizing the soybean root was quantified as CFU per gram of fresh root weight (n = 3 for each treatment).

### Dual inoculation efficacy between SG09 and PGPB in response to salinity

To assess the superior effects of the dual inoculation of SG09 with either OFT2 or OFT5 under saline conditions, the recovery of soybean plants after salinity stress was examined under greenhouse conditions. Plants inoculated with SG09, SG09 + OFT2, or SG09 + OFT5 were exposed to 60 mM NaCl for 12 days, and then non-saline B & D nutrient solutions were applied until harvest. Plants inoculated with SG09 alone, without salinity treatment, were used as positive controls. The pots were arranged in a randomized complete block design with four replicates, with each replicate representing two plants. The procedure for the dual inoculation and growth conditions for soybean seedlings was the same as described above. Eight weeks after sowing, the plants were harvested, and plant biomass, photosynthesis traits, nodulation, and N_2_-fixation were measured.

### Statistical analyses

All experiments followed a randomized complete block design with four replicates. The data were subjected to statistical analysis using one-way analysis of variance (ANOVA), and mean values were compared using Duncan’s Multiple Range Test (DMRT) at a significant level of p < 0.05.

### Bioethical statement

We confirmed that all local, national, or international guidelines and legislation were adhered to for the use of plants in this study (https://www.nature.com/srep/journal-policies/editorial-policies#research-involving-plants).

### Supplementary Information

Supplementary Information.

## Data Availability

All data generated or analyzed during this study are included in this published article and its supplementary information files.
