# Supplementary material for: Synergistic N2-fixation and salt stress mitigation in soybean through dual inoculation of ACC deaminase-producing Pseudomonas and Bradyrhizobium
Source: Sci Rep. 2023 Oct 10;13:17050. doi: 10.1038/s41598-023-43891-4 (PMC10564950; doi:10.1038/s41598-023-43891-4)
Supplement: Supplementary file 1 — Supplementary Information. [file 41598_2023_43891_MOESM1_ESM.pdf]

Dual inoculation of ACC deaminase-producing *Pseudomonas* and *Bradyrhizobium*: A synergistic approach to N_2-fixation and salt stress mitigation in soybean

Win et al.

Supplementary Table S1. Nodulation of non-inoculated rhizobial plants grown in 0 and 60 mM NaCl

	Nodule No.	Nodule DW (mg)
Un-inoculation	7.8 ± 1.7	10.3 ± 5.4
OFT2	8.0 ± 3.2	9.5 ± 5.8
OFT5	6.3 ± 1.3	8.5 ± 5.4
Un-inoculation+ NaCl	Not detected	Not detected
OFT2 + NaCl	Not detected	Not detected
OFT5 + NaCl	Not detected	Not detected

Supplementary Table S2.

Shoot micro-ions uptake in non-rhizobia inoculated and dual inoculated with rhizobial strains in soybean plants grown under 0 and 60 mM NaCl. Values are presented as means (S.D.), *n* = 4. Different letters indicate classes that show significant differences ($p < 0.05$) using Duncan's Multiple Range Test (DMRT) ($p < 0.05$).

Traits		B		Mn		Zn	
Inoculation/treatments		0 mM	60 mM	0 mM	60 mM	0 mM	60 mM
Non-Rhizobium	un-inoculation	4.72 ± 0.39ab	4.00 ± 0.27c	9.42 ± 1.27a	6.14 ± 0.08c	0.63 ± 0.06a	0.70 ± 0.06a
	OFT2	4.89 ± 0.43a	4.24 ± 0.25bc	8.13 ± 0.76ab	7.15 ± 1.23bc	0.72 ± 0.07a	0.72 ± 0.07a
	OFT5	4.61 ± 0.21ab	4.29 ± 0.37bc	6.75 ± 0.52bc	6.92 ± 0.84bc	0.71 ± 0.08a	0.71 ± 0.05a
Rhizobium	USDA110	4.01 ± 0.40bcd	2.68 ± 1.20ef	6.67 ± 1.00cdef	4.80 ± 2.00ef	0.42 ± 0.04a	0.47 ± 0.15a
	110+OFT2	4.29 ± 0.90abc	3.04 ± 0.71def	8.06 ± 1.48cd	5.74 ± 1.11def	0.45 ± 0.09a	0.48 ± 0.05a
	110+OFT5	4.75 ± 0.54ab	2.10 ± 0.54f	10.93 ± 2.33a	4.35 ± 1.08f	0.52 ± 0.06a	0.39 ± 0.09a
	SG09	3.89 ± 0.38bcd	3.31 ± 0.49cde	8.46 ± 1.32bc	6.55 ± 1.45cdef	0.40 ± 0.03a	0.5 ± 0.07a
	SG09+OFT2	5.06 ± 0.20a	4.15 ± 0.23abc	10.77 ± 2.11ab	8.09 ± 0.20cd	0.52 ± 0.02a	0.56 ± 0.03a
	SG09+OFT5	4.86 ± 0.80ab	3.60 ± 0.38cde	8.54 ± 1.45bc	7.14 ± 1.36cde	0.53 ±0.10a	0.48 ± 0.06a

Supplementary Table S3. Effects of different NaCl levels on the growth of two *Bradyrhizobium* strains.

Rhizobium	NaCl (mM)	OD value	% of decrease
USDA110	0	0.36 ± 0.01a	
	60	0.10 ± 0.04b	72%
	100	0.05 ± 0.03c	86%
SG09	0	0.61 ± 0.03a	
	60	0.51 ± 0.04b	16%
	100	0.06 ± 0.05c	90%

Note: Flasks containing the USDA110 and SG09 strains were separately incubated onto liquid TY an HM medium on a rotary shaker at 28◦Cand 180 rpm until the absorbance reached 0.4 at 600 nm. Subsequently, 50µl of each culture was added to 15 mL of fresh liquid medium in a flask containing 0, 60 mM, and 100 mM NaCl and further incubated at 28 ◦C and 180 rpm. The optical density (OD) of the cultures was recorded 6 days at 600 nm. Each treatment had six biological replicates. An individual flask was considered one biological-replicate.

Supplementary Figure. S1

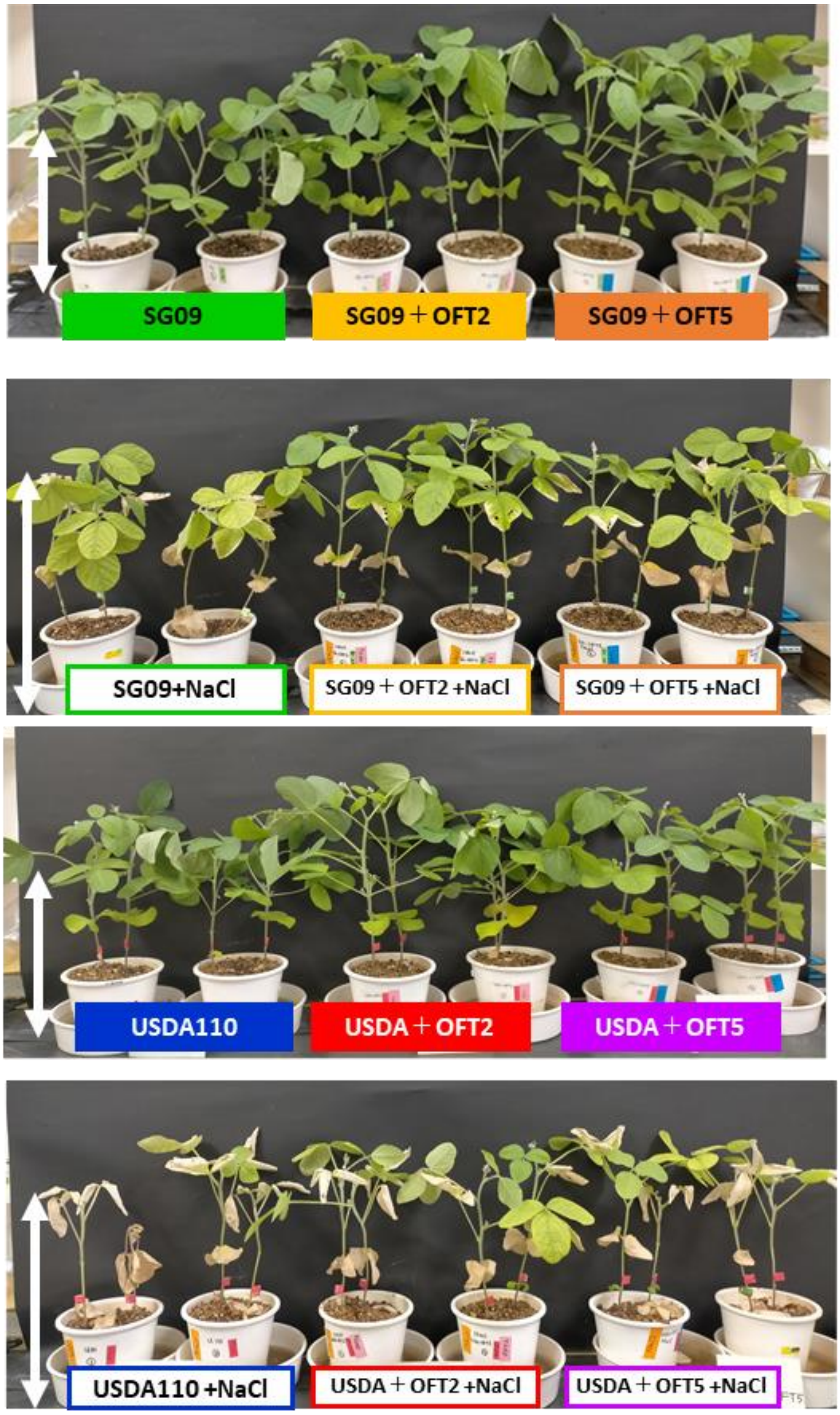

Supplementary Figure 1. Growth-promoting effects of dual inoculation of *Pseudomonas* bacteria (OFT2 and OFT5) expressing 1-amino-cyclopropane-1-carboxylate (ACC deaminase with two rhizobial strains of USDA110 and SG09 in soybean grown under normal and 60 mM of NaCl stress for 21 days. Scale bar: 15 cm

Supplementary Figure. S2

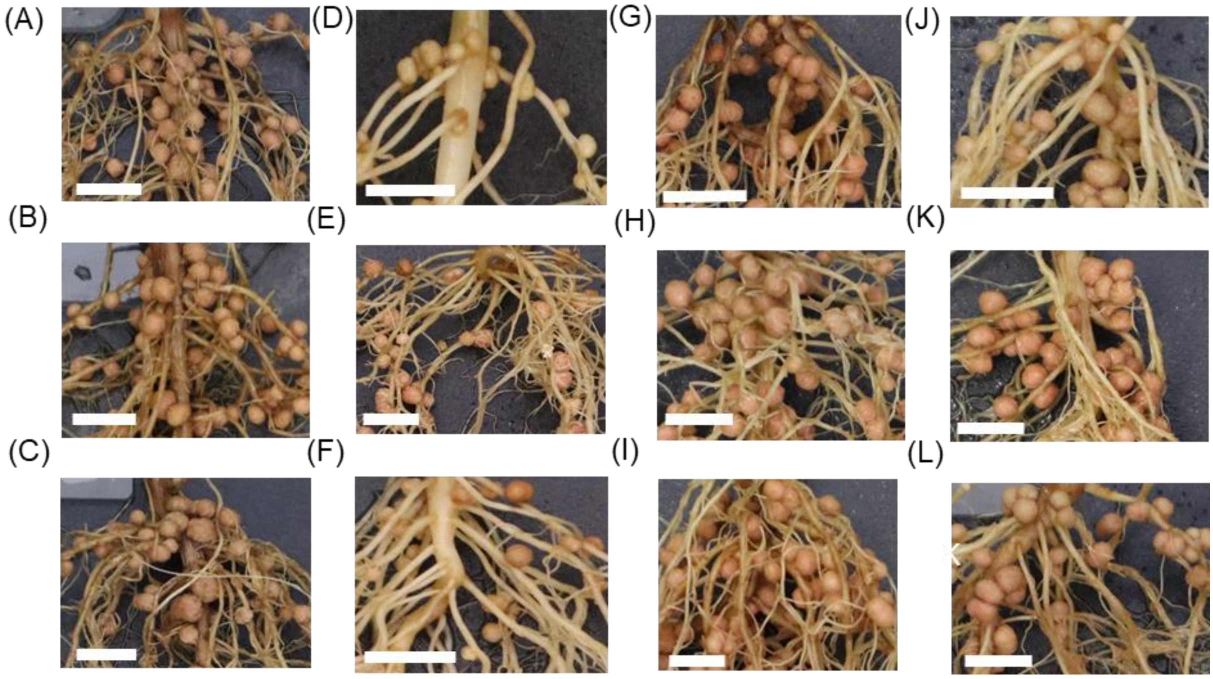

Supplementary Figure 2. Effect of PGPB on nodulation of soybean plants (A) USDA110, (B) USDA110 + OFT2, (C) USDA110 + OFT5, (D) USDA110 + NaCl, (E) USDA110 + OFT2 + NaCl, (F) USDA110 + OFT5 + NaCl, (G) SG09, (H) SG09 + OFT2, (I) SG09 + OFT5, (J) SG09 + NaCl, (K) SG09 + OFT2 + NaCl, and (L) SG09 + OFT5 + NaCl. Scale bar: 1 cm
